# An appraisal of the implementation of the national school feeding programme and its effect on enrolment and attendance in public primary schools in Southeast, Nigeria: perception of heads of schools

**DOI:** 10.1186/s40795-023-00695-z

**Published:** 2023-03-02

**Authors:** Chibuike Innocent Agu, Edmund Ndudi Ossai, Onwe Emeka Ogah, Ifunaya Clara Agu, Ifeyinwa Akamike, George Onyemaechi Ugwu, Nwobodo Edwin, Blessing Lucy Ewenyi, Benedict N. Azuogu

**Affiliations:** 1 Health Policy Research Group, College of Medicine, University of Nigeria Enugu-Campus, Enugu, Nigeria; 2 Department of Community Medicine, Alex-Ekwueme Federal University Teaching Hospital, Abakaliki, Ebonyi Nigeria; 3 Department of Paediatrics, Alex-Ekwueme Federal University Teaching Hospital, Abakaliki, Ebonyi Nigeria; 4 Department of Obstetrics and Gynaecology, College of Medicine, University of Nigeria, Enugu Campus, Nigeria; 5 Department of Physiology, College of Medicine, Nnamdi Azikiwe University, Awka, Nnewi Campus, Nigeria

**Keywords:** NHGSFP, Implementation, Public primary schools, Qualitative study, Enugu, Nigeria

## Abstract

**Introduction:**

The National Home Grown School Feeding Programme (NHGSFP) was re-launched in Nigeria in 2016, eleven years after it was first introduced in the country, with Enugu as one of the beneficiary States. The objectives of the programme are to improve the health of school children and aid in the realization of Universal Basic Education (UBE) goals. This study explored the opinions of heads of public primary schools on the implementation and policy benefits of NHGSFP in Enugu, southeast Nigeria.

**Methods:**

This was a cross-sectional study conducted among 24 headmasters and headmistresses purposively selected from public primary schools in the Enugu metropolis. Qualitative data were collected through the use of a pretested Key Informant Interview (KII) guide, and analyzed using a thematic approach.

**Results:**

All the participants were aware of the NHGSFP, which involved the provision of one mid-day meal per child per school day to the pupils, and all their schools were part of the programme. Most of the participants complained about the nutritional quality and quantity of the school meals which they felt were poor. None of the schools had a kitchen within the school premises, and all the participants admitted that deworming was not regularly carried out, as part of the programme. Most of the participants believed that the objectives of the feeding programme, including, reduced hunger among learners, increased school enrolment, attendance and enhanced participatory learning, were being met.

**Conclusion:**

Although the NHGSFP was implemented in every school in Enugu metropolis, Enugu State, Nigeria, regular deworming of pupils was not carried out, and there were concerns about certain aspects of the implementation, such as inadequate funding and poor quality of school meals. Thus, there is a need for the introduction of deworming and more allocation of funds to the programme to improve the quantity and nutritional quality of school meals.

**Supplementary Information:**

The online version contains supplementary material available at 10.1186/s40795-023-00695-z.

## Introduction

The problem of malnutrition remains severe, and many children around the world are already stunted, underweight and/or suffering from multiple micronutrient deficiencies by the time they start school [1]. Most of these children come from developing countries, where the problem is even more pronounced [2]. In low and middle-income countries, child under-nutrition contributes to about 45% of under-5 child mortality [3]. About 66 million school-age children in the developing world go to school every day on an empty stomach, and hungry children become easily distracted and have difficulties concentrating in their studies while in school [4–6]. In addition, low school enrolment, high dropout rates and poor educational outcomes, among other factors, have been linked to hunger [7]. Evidence shows that only three countries-India, Nigeria and Pakistan- are home to approximately half (47.2%) of all stunted children, with Nigeria having the second-highest burden in the world [8]. The country, also, has about 3.4 million children who are wasted [8]. These conditions have long-term negative effects on individuals and societies including poor cognitive development, lowered performance in education and low productivity in adulthood [9, 10].

Efforts to address nutritional challenges include the school feeding programme which has its origins in the 1930s when it was introduced in the United Kingdom (UK) and the United States (US) with the express purpose of enhancing children’s growth [2]. On its part, the Federal Government of Nigeria showed political commitment to tackling the current nutritional situation of the country by re-launching a national home-grown school feeding programme in 2016, eleven years after it was first introduced in the country with support from the New Partnership for Africa’s Development (NEPAD) and the United Nations Children’s Education Fund (UNICEF) [11]. Enugu State is one of the States selected to begin a phased–pilot rollout of the school feeding programme. The goal is to provide one nutritionally adequate meal each school day to all primary school pupils in the country. The objectives of the national program are to improve the health of school children, increase enrolment, attendance, and retention, as well as completion and learning achievements among pupils, thereby contributing to the realization of the goals of Universal Basic Education (UBE) [12]. The meals should be prepared from locally sourced or procured food items or farm produce grown by smallholder farmers, with necessary fortification/supplementation, and regular deworming of pupils. Thus, farmers are to benefit from improved access to school feeding markets, while communities take advantage of the new opportunities across the supply chain, such as catering, processing and food handling jobs, leading to a boost in the local economy. As a food-based safety net programme, it is also intended to help promote food security in the beneficiary households.

According to the implementation guidelines on the national school health programme, for effective implementation of the school feeding programme, each school is expected to have an appropriately sited and well-equipped kitchen, and facilities for safe waste disposal within the school premises and school farms or gardens [13]. There should also be adequate sanitation and hygiene practices as well as routine medical examinations and vaccination of food handlers [13].

Although the need for school feeding programs is still contentious on theoretical, political, and implementation levels, its proponents cite a number of logistical, empirical, and ethical considerations [2]. For instance, the school is, in principle, an important setting where health and education interventions, such as a feeding programme can be implemented, because they are available even in rural areas, and most children pass through them [14]. Up to 61% of children between the ages of 6–11 years regularly attend primary school in Nigeria [15]. Moreover, school feeding programmes (SFPs) generally improve educational outcomes by increasing learners’ years in school, especially for young girls [2, 7]. Every additional year of education for a girl results in a 5–10% reduction in her offspring’s mortality.

Most of the studies on the Home Grown School Feeding Programme have focused on other aspects of the programme and seemed to have left out the opinions of headmasters or headmistresses of primary schools. However, in view of their positions as the administrative heads of primary schools, it is important to ascertain their views on the effectiveness of the programme‘s implementation toward the realization of its core objectives. Thus, this study was designed to assess the implementation and policy benefits or otherwise of the programme in public primary schools in Enugu, Enugu State, Nigeria, from the standpoint of school administrative heads. The study explored their opinions and perceptions in this regard, and there is no doubt that the findings can generate evidence which will contribute to the improved implementation of the national programme.

## Conceptual framework

**Fig. 1:**
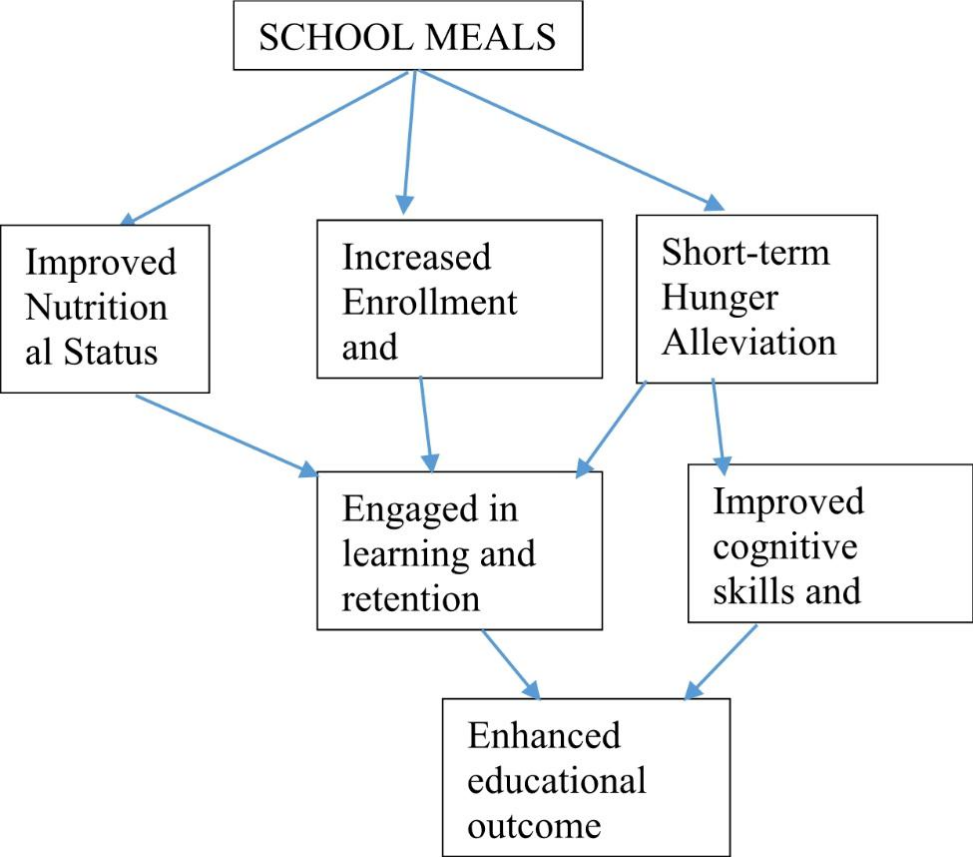
Conceptual framework on school feeding programme [16]

There are three pathways through which a school feeding programme can contribute to educational improvements for children [16, 17]. First, the programme can increase enrolment and attendance at school by providing incentives for poor families to send their children to school and keep them there [18, 19], (Fig. 1). The second is the alleviation of short-term hunger, which enhances their cognitive abilities and attention span. The third route involves enhancing children’s nutritional status by giving them more calories and nutrients in addition to their regular diet. Also, according to Maslow’s Hierarchy of needs theory, food is a basic physiological need and higher-order requirements can only be satisfied if the basic ones are addressed. Thus, for effective learning activities, learners’ physiological demands must be satisfied for them to focus on learning and for educational institutions to accomplish high-quality learning [20]. Therefore, improved nutrition indirectly raises academic achievement by encouraging children’s attendance to and focus in school [16].

## Methods

### Study Design and Area

The study was a cross-sectional study which explored the perspectives of heads of public primary schools on the implementation of home-grown school feeding programme in public primary schools in Enugu Metropolis. The study was conducted between August and October 2020 in Enugu State, one of the five states in the southeast geo-political zone of Nigeria. The capital city of Enugu has an estimated population of 773,000 people based on the same census [21]. It consists of urban parts of three local government areas, including Enugu East, Enugu North and Enugu South LGAs. The vegetation of the state is primarily of the forest type, although it extends into the Savannah on the state’s northern borders. It has separate wet and dry seasons and an average yearly rainfall of 1536 mm [22]. Agriculture is the primary occupation of the people, and the major crops grown include cassava, yam, cocoyam, vegetables, and oil palm. Chicken, goats, sheep, and cattle make up most of the animals reared [23]. Other livelihood activities in the State are fishing and wine tapping.

The Nigerian formal educational structure is divided into, basic education, senior secondary school education and tertiary education. This is based on the 9-3-4 formula, which means 9 years of basic education (made up of 6 years of primary school for children of ages 6 to 11 years, and 3 years of Junior Secondary School for children of ages 12 to 15 years), 3 years senior secondary school and 4 years tertiary education (including universities, polytechnics and colleges of education) [24]. The focus of the study was the public primary schools, and there are 117 public primary schools are located in the capital city of Enugu.

### Study participants

The study participants comprised administrative heads of the 24 selected public primary schools in the Enugu metropolis, Enugu State, Nigeria. All the headmasters or headmistresses of selected public primary schools in the urban area of the three LGAs making up the Enugu metropolis were included in the study. However, heads of public primary schools who had not worked in that capacity for up to one month were excluded from the study as this was considered enough period for them to know much about the feeding programme.

### Sampling technique and study instrument

The number of public primary schools in Enugu urban was 117, consisting of 27 in Enugu south, 36 in Enugu East and 54 in Enugu North. This study adopted both random and purposive sampling techniques to recruit participants. To ensure proportional representation of the LGAs, the schools were selected in the ratio of 6: 7: 11 public primary schools respectively, making a total of 24 public primary schools, while the heads of the schools were purposively interviewed for the study until data saturation was reached. Qualitative data were collected using a key informant interview (KII) guide, which was pretested in two public primary schools located in the capital city of Ebonyi State. The KII guide was from the National School Health Policy [12] and the review of relevant literature [25, 26]. Questions were asked in different areas including awareness, and availability of the school feeding programme, quality and quantity of the school meals, sources, packaging, storage and processing of food items, and food fortification.

### Data collection method

This was carried out between August and October 2020 by the researcher and with the help of trained research assistants. Appointments were arranged with the participants by the researcher for the interviews. Interviews were tape-recorded with the permission of the individual participant. Each interview was conducted in venues that were convenient for the participants’, such as the participant’s office. The use of face masks, social distancing and other Nigeria Centre for Disease Control (NCDC) guidelines on Covid-19 prevention [20] were strictly adhered to. All interviews were done in English language and lasted about 45 min for each session.

### Data analysis

Tape-recorded interviews were transcribed verbatim after carefully listening to the tapes. The scripts were compared with the written notes for completeness and accuracy, and each script was checked against the audiotape by an independent reviewer. Then, the transcripts were read by two independent researchers in order to get a thorough overview and gain a full insight into the data. Starting with the richest ones, the transcript of every interview was studied thoroughly and coded. From the codes, patterns were identified and initial themes were generated. Following research team meetings, the themes were reviewed, refined and finalized. Four themes emerged, and they include awareness of the implementation of home-grown school feeding programme, quality and quantity of school meals, facilities and personnel for effective implementation of home-grown school feeding and the effect of the programme on school enrolment, attendance and participatory learning among pupils.

### Rigour and trustworthiness

To ensure rigour in this study, we employed trustworthiness criteria of credibility, confirmability and transferability [27]. The findings of the study were presented to the interviewees in the process of participant validation (member-checking) and review to ensure their credibility. Two independent researchers went through the transcripts and the findings and provided feedbacks, while team meetings provided opportunities for peer debriefing. Lastly, by writing the findings of our study using the participants’ words, we provided the reader with context and thick rich description to meet the transferability criterion [28].

## Results

### Participants’ profile

The age range of the participants was 49 to 60 years and a median age of 53.5 years. Most of them (23) were female. All the participants had tertiary level of education, and had all served as the headmaster or headmistress in their various schools for more than one year.

### Awareness of the implementation of the national home-grown school feeding programme

All the participants were aware of the national home-grown school feeding programme and their schools were part of it. It involves the provision of one mid-day meal per child per school day to the pupils. However, all the participants lamented that the programme did not cover the entire primary schools in the State. Also, in the schools were it was being implemented, not all the pupils were covered as only primaries 1 to 3 were included in it. Some of them made their minds known in the following quotes:

*“I am aware of the school meal [school feeding programme], which I think started last year or last two years, and my school is involved in it. However, it’s only for pupils in primary 1 to primary 3 with the exclusion of those in classes 4 to 6.”* (SHEN02_Headmistress)

*“Yes, school meals are provided for the pupils in some schools, including ours, by the federal government. Food vendors do come and give from primary 1 to 3, food every day.”* (SHEN04_Headmistress)

The exclusion of some of the pupils from the programme did not go down well with most of the participants, with some of them calling on the authorities to expand the programme to include school children in classes four to six. Two of the school heads expressed themselves thus:

*“As you know, it is only for those in primaries 1 to 3. I sincerely plead with government authorities to extend it to those in primary four to six”* (SHEN02_Headmistress)

*“I think the school feeding programme is a wonderful idea, but it is not covering the entire pupils, so I call on the government to do the needful by extending to those in primaries 4 to 6 as that will go a long way to help in realizing the aim of the programme.”* (SHEE04_Headmistress)

### Quality and quantity of school meals provided for pupils

While most of the participants complained about the quality of the meals which they felt were poor, all of them spoke maintained that the quantity of the meals was abysmally poor. Some of the typical responses from them include;

*“The challenge is that the food is not enough. They employed food vendors that are not giving the children adequate food. The food vendors themselves complain that they are being paid poorly and that they cannot use meager amount to provide enough meal for the children “*(SHES01_Headmistress)

*“Concerning the quality, I don’t think that they (vendors) are even trying to make the food balanced at all. The type of food they prepare is so poor in quality. When you taste it during supervision, it is so tasteless!”* (SHES03_Headmistress)

One of the participants tried to offer an explanation for the poor quantity and quality of the meals. She attributed it to poor funding of the programme, pointing out that an increase in funding may lead to an improvement in the school meals.

*That’s what I am complaining about; the meal is very poor and so small. I know they are being given only seventy naira (70) for each meal, but if they can make it up to hundred naira (100) per meal per child it will be better”* (SHEN05_Headmistress)

Few participants thought that the quality could be managed, even though the meals are so small in quantity. A participant noted that there are times when the meals are insufficient to go round all the eligible pupils. In her remark, she had this to say:

*“The quality is a bit good but the quantity is too small. At times it doesn’t go round; with respect to the quantity they don’t get it right at all, although it is still better than having no food.”* (SHEE06_Headmistress)

There was a consensus, among all the participants that the meals served to the pupils by the food vendor were sourced locally as recommended in the implementation guideline on National School Health programme. Two of the responses from the participants are expressed in the following quote:

*“Yes, the materials are from our local environment; for instance sometimes they prepare “igba-oka” or “okpa” [local food delicacies], which are wrapped with local leaves.”* (SHES02_Headmaster)

*“They (food vendors) do give them local meals such as “igba-oka” [local food delicacies prepared from bambara beans]. They also use vegetables which I believe they get locally for the preparation of some of the meals.”* (SHEE01_Headmistress)

### Facilities and personnel for the effective implementation of NHGSFP

None of the schools had a kitchen within the school premises as disclosed by each of the participants. This is a condition which they all complained about. One of their views is presented in the following quote:

*“They [food vendors] lack a place (kitchen) to be cooking; they have been troubling me to give them a place to be cooking, but there is no space for that*.”(SHES02_Headmaster)

The absence of kitchen facilities in the schools for preparing school meals affected the effectiveness of the teachers’ food monitoring activities. This is because this robbed them of the opportunity to monitor how the meals are prepared closely as indicated in the following quote.

*“We don’t have a kitchen for preparing school meals here and as a result, we find it impossible to monitor what they do, how they prepare the food” “The government should really look into the issue of where to be cooking the food and try and help the vendors. Making a provision for them to be cooking the food in school will go a long way in helping them”* (SHES03_Headmistress)

Few of the participants, however, opposed the idea of siting a kitchen facility within the school premises, pointing out that such an act would not argue well with the learning activities of the pupils. A female participant who expressed her opinion noted that preparing the meals in schools could be distracting to the pupils. In her words,

*“The school does not have kitchen facilities, and in fact, there is no space for that. So, how can children be in the class and be perceiving the aroma of food? It will be disturbing them!”* (SHES04_Headmistress)

A few of the participants, also, expressed concern over the hygiene practices of the vendors/food handlers, noting that these could be compromised as the meals are prepared away from the watchful eyes of the school authorities. They stated that since the meals are prepared outside the school environment, they are hampered from observing the sanitary and hygiene practices of the vendors effectively.

A male participant expressed herself thus:

*“One major problem thrown up by the fact that the school doesn’t have kitchen for the food preparation is that we cannot monitor the environmental condition of where the food is prepared, how clean, etc. It is possible that they do not maintain good sanitary practices while cooking”* (SHES02_Headmaster)

Also, one of the participants who shared their opinions noted that some parents prevented their wards from taking the meals because they were not sure of how they were prepared.

*“Where they cook the school food may not be hygienic, and so, some parents don’t want their children to take it because they don’t know where they cook the food and how clean the place is,”* (SHEE03_Headmistress)

Concerning deworming, all the participants admitted that, although deworming took place in their schools, it was not on a regular, three-monthly basis as stipulated in the implementation guidelines of National school health programme, and not as part of the NHGSFP. In the words of a participant:

*“Deworming of our school children is done occasionally, not as part of the feeding programme, but, by the health officials of local government health center through the help of some NGOs.”* (SHEN07_ Headmistress)

One participant indicated that as a result of the irregularity of deworming exercise, their school sometimes advised parents to deworm their wards by themselves. In her remark, she had this to say:

*“No we don’t do that (deworming) often. The last time we dewormed them was over a year ago and it was organized by the old boys’ association of the school. But what we often advise parents to deworm their children by themselves.* (SHES05_Headmistress)

Another participant admitted that vitamin A supplementation was not done as part of the home-grown feeding programme. In her words,

*“No, they don’t give vitamin A supplementation; the only thing is that vendors come and give them food, but nothing for vitamin A”* (SHEN07_Headmistress)

### Effects of NHGSFP on school enrolment, attendance and participatory learning

Most of the participants believed that the objectives of the feeding programme, such as, reduced hunger among learners, increased school enrolment, attendance, and enhanced participatory learning, were being realized. A participant described the effects of the programme on hunger reduction thus:

*“It [the feeding programme] is very important in hunger reduction because I could remember that before it started it, in 2015 when I came here newly, you would see a child crying because he did not eat, but it no longer happens again. This is because school meal is served on daily basis and the children now eat while in school, so it is very important in reducing hunger among the pupils”* (SHEN07_Headmistress)

As regards school enrolment, it was noted that there was an improvement in school enrolment. Some of the participants who made their views known observed that as the information spread that pupils were fed in schools, more pupils were enrolled in schools where school feeding programme is implemented. A participant expressed her thought in the following quote:

*“We noticed that when people who are not in school hear that those who come to school have school meal they are encouraged to go and enroll in a school, so that they will also enjoy the meal.”* (SHEE04_Headmistress)

Another participant claimed that pupils in private primary schools, which are not part of the feeding programme, had to change to public school in order to benefit from programme.

*“I believe that people get registered in primary schools a result of the school meal programme, because even some pupils in private schools had to change to public schools when they learnt about the school meal.”* (SHES06_Headmistress)

School attendance was noted to have been enhanced by the school feeding programme. Some of their comments were captured as follow:

*“Yes it [the school feed programme] increases their attendance to school; it inspires them to come to school. Even when they feel like staying at home and they remember they will be fed in the school they will now join others and come to school.”* (SHEE04_Headmistress)

*“… I can say it enhances school attendance because there are few pupils in my class that now come to school regularly and one of the parents confided in me that the school feeding is instrumental to that as they used to find it hard providing food for their children.* (SHEN01_Headmistress)

However, a participant was of the opinion that school feeding programme did not in any way enhance school attendance given that not all parents permitted their children to take the school meals. The participant who expressed herself, stated that they had not observed any increase in school attendance. According to her:

*“It does not enhance their coming to school because we have not noticed any increase in the number of people who come to school. Besides, a lot of parents tell their children not to take the school meal because they may start purging after eating it. So, how can it now make them to come to school?”* (SHEN08_Headmistress)

On the contrary, there is an agreement among the participants that participatory learning is positively affected by the feeding programme. Some of them who spoke expressed themselves in the following quotes:

*“At least some of the kids who never fed at home used to feel tired when you are talking to them, and will simply not be interested in classes, but once you give them food, they will show interest in learning.”* (SHEN04_Headmistress)

*“…we discovered that some of the pupils will be feigning sick and show no interest in what is going on in the class, but if you get food, such as “okpa” [local food delicacy prepared from bambara beans] and give to them, they will be revived and take part in learning and other class work. That is to say that their problem may have been hunger abinitio.”* (SHEN07_Headmistress)

## Discussions

This study explored the views and opinions of public primary school headmasters and headmistresses on the effective implementation of the national home grown school feeding programme and its effect on school enrolment and attendance in Enugu, Enugu State. The results showed that NHGSFP was implemented in all the schools, but deworming, - a critical component of school feeding programme - was missing. There were concerns about the poor financing of the programme, inadequate infrastructure as well as the poor quality and quantity of the school meals.

It is not surprising that all the participants were aware of the existence of the programme, since it was implemented in all the schools. A study in Osun State, southwest Nigeria, also, reported that the majority of the respondents knew about the school feeding programme [11]. The finding might be an indication of the widespread nature of the programme, being one of the key elements of the social investment programme (SIP) of the government of Nigeria. Currently, the program benefits over nine million students from 54,619 schools, with the involvement of 80,000 farmers and over 102,097 cooks in 26 states across the nation [29]. Likewise, the finding that the programme was being implemented in all the public primary schools is in keeping with the reports from earlier studies in other parts of the country [5, 11, 19].

This study indicated that most of the participants decried the quality and quantity of the meals provided for pupils as poor, although they were largely sourced or prepared from local food materials. Similarly, a study in Ghana documented that foods served to pupils were insufficient in quantity and quality. This negates the programme’s goal of achieving food security, which is about having access to food that is both sufficient in quality and quantity in a socially and culturally acceptable manner [2, 13]. To tackle the challenges of poor quality of school meals, the World Health Organization (WHO) recommended the implementation of nutrition policies which should effectively control the types of food available in schools to enhance children’s nutrition [30, 31]. Countries, such as Australia and Iran have adopted such policies, with good results and positive impacts on children’s diet [31]. Additionally, a recent call has been made for the establishment of school farms and legal nutritional regulations to supplement governments’ contributions and check the nutritional quality of school meals respectively [29].

The poor state of the meals may be attributed to poor funding with a budget of a paltry sum of seventy naira (70.00) per day for a child, which was observed from the study. This is a far cry from the average expenditure on individual school meal in some developed nations [32]. This may also explain why the programme is only available to pupils in public primary schools, classes 1 to 3 which is an emerging system of SFP that excludes some classes of pupils in schools where the programme is implemented. However, given the advantages of the programme, as shown in this study, it is crucial that the feeding programme be expanded to, also, cover higher classes in public primary schools. Thus, the governments need to make policies to incorporate pupils in public primary schools, classes 4 through 6 in the SFP.

This study also showed that deworming is not carried out in public primary schools as part of the school feed programme, and this affirms the result of similar studies conducted in South West, Nigeria and Ghana [25, 33]. The omission is unfortunate as children are particularly susceptible to parasitic infestations, no matter how well fed. When left untreated, worm infestations prevent children from growing normally, lead to poor nutrition, and impair their ability to focus and learn. On the other hand, regular treatment helps school-age children maintain optimal health and nutrition, which increases enrolment and attendance rates, fewer missed classes, and greater academic performance [34]. Thus, it is crucial that school-age children are regularly dewormed as part of the school feeding program to ensure the success of the initiative.

The finding of non-availability of kitchen facilities in all the schools means that the meals are not prepared in the school environments, but are contracted out to food vendors. Consequently, the process of food preparation and storage are not supervised effectively by the school authorities, and this could lead to compromised sanitary and hygiene practices by the food vendors. This is in contrast to the result from a study in Ghana which reported that communities, in conjunction with the school’s Parent Teacher Association (PTA), provided kitchens and storehouses in schools for the implementation of the feeding programme. In general, catering services differ among countries. For instance, while the majority of the schools in Rome, Italy, have meals prepared in school kitchens, catering services for schools are typically outsourced to private companies in Spain [32]. These variations could be the result of cultural and economic disparities across the nations.

Despite the above drawbacks, it is pleasing to note that most of the participants believed that the objectives of the feeding programme, such as reduced student hunger, increased school enrolment, attendance and retention were being realized. This confirms the findings from previous studies both in Nigeria and other countries that HGSFP has a positive impact on the educational outcomes of pupils, although the benefits are more in low-income countries [29 , 35, 36]. The introduction of the School Feeding Programme has, indeed, made a difference in developing countries, by causing an increase of 30% in pupil enrolment in schools [35]. In a similar vein, different studies in Abuja, Northcentral, Abakaliki, Southeastern and Osun south-west, Nigeria observed that SFPshave helped in increasing the number of students enrolled in schools, encouraging regular attendance, improving punctuality, and lowering the dropout rate [29, 35, 37]. Also, in countries, such as Brazil, Philippines, Cambodia, Mali, El Salvador, Indonesia, Ghana, Bangladesh, and Ecuador, SFPs have increased enrolment and attendance rates over the years [37, 38].

The study is limited by the fact that the pupils who are end users of the NHGSFP and policy-makers who oversee its implementation were not included in the study. However, the use of KII enabled an in-depth exploration of the perspectives of public primary heads in the State, while data synthesis was based on guidelines on the national school health programme and the theoretical framework. Future research should explore the views of pupils and the impacts of the feeding programme on their general nutritional status and cognitive development. Also, such studies should investigate the effect of the programme on food security in the beneficiary households.

## Conclusion

The national home-grown school feeding programme was implemented in every school in the Enugu metropolis, Enugu State, Nigeria. However, deworming, which is a vital part of school feeding programmes was missing. There were, also concerns about the programme’s weak funding, inadequate infrastructure, monitoring of food preparation, and the poor quality and quantity of school meals. Thus, there is a need for the regular deworming of pupils as part of the programme; the government should allocate more financial resources for the effective implementation of the programme, while efforts should be made by schools to establish school farms in order to supplement food supply.

## Electronic supplementary material

Below is the link to the electronic supplementary material.

Supplementary Material 1

## Data Availability

The dataset used for this study is available and can be obtained from the corresponding author upon request.
