# Supplementary material for: An appraisal of the implementation of the national school feeding programme and its effect on enrolment and attendance in public primary schools in Southeast, Nigeria: perception of heads of schools
Source: BMC Nutr. 2023 Mar 2;9:37. doi: 10.1186/s40795-023-00695-z (PMC9979116; doi:10.1186/s40795-023-00695-z)
Supplement: Supplementary file 1 — Supplementary Material 1 [file 40795_2023_695_MOESM1_ESM.docx]

**Implementation of the National School Feeding Programme and its effect on enrolment and attendance in public primary schools in Southeast, Nigeria**

**KEY INFORMANT INTERVIEW GUIDE**

1. Are you aware of the national home-grown school feeding program? (i. Yes, ii. No). If yes, can you tell us what you know about it? Is the feeding programme being implemented in your school? When was it started in your school? By whom? Which class of pupils benefits from the programme?
2. What is your opinion about the quality of the school meals? How about the quantity?
3. Are school children provided with nutritionally adequate meal each school day? Please, explain your response
4. Are the meals prepared from food items produced or sourced packaged, processed, stored and utilized locally? (i. Yes, ii. No) Please explain your response
5. In your opinion, what do the meals/diets comprise of? (**Prompts:** carbohydrate, beans, soya beans, eggs, fresh milk, yoghurt, cocoa drink, etc?) pls, explain your answer
6. Is a kitchen (Kitchen equipment? Storage space? ) located within the school premises? (i. yes; ii. No)
7. If No, where are the meals prepared?
8. What is your opinion about where the school meals is prepared? (**Probe:** on monitoring and supervision of meal preparation by the teachers, hygiene practices of the school meal vendors)
9. Does your school have farms and gardens? Do you get fresh fruits or vegetables that are grown in the farms or gardens?
10. Is there regular de-worming exercise at least once every 3 months? (i. Yes, ii. No). If not, how often do you have it?
11. Are food vendors certified before they are engaged? (by who?, medical check. How often?)
12. If yes, how often are they re-accessed?
13. Could you tell us what you think are the effect of the feeding programme on your school? Probe the following:
14. School enrolment
15. School attendance
16. participatory learning in classes
17. In your opinion, what are the challenges to the effective implementation of the school feeding programme?
18. What is/are your recommendation(s) on the way forward?
